# Host-feeding behaviour of *Dermacentor reticulatus* and *Dermacentor marginatus* in mono-specific and inter-specific infestations

**DOI:** 10.1186/s13071-015-1078-9

**Published:** 2015-09-17

**Authors:** Alicja Buczek, Katarzyna Bartosik, Zbigniew Zając, Michał Stanko

**Affiliations:** Chair and Department of Biology and Parasitology, Medical University of Lublin, Radziwiłłowska 11 St. 20-080 Lublin, Lublin, Poland; Institute of Parasitology, Slovak Academy of Sciences, Hlinkova 3, 040 01 Košice, Slovak Republic; Institute of Zoology, Slovak Academy of Sciences, Dŭbravská cesta 9, 845 06 Bratislava, Slovak Republic

**Keywords:** Dermacentor reticulatus, Dermacentor marginatus, Host-feeding behaviour, Co-feeding ticks

## Abstract

**Background:**

Given the sympatric occurrence in some regions of Europe and the great epidemiological significance of *D. reticulatus* and *D. marginatus* species, we investigated the behaviour of these ticks during inter-specific and mono-specific host infestations.

**Findings:**

The investigations were conducted on rabbits at 20 ± 3 °C and humidity of 38 ± 1 %. The inter-specific infestations groups consisted of 20 females and ten males of *D. marginatus* and 20 females and ten males of *D. reticulatus* on each host, whereas mono-specific infestations involved 40 females and 20 males of each species.

The investigations have demonstrated competition between the two tick species resulting in modification of the behaviour on the host and the feeding course in *D. marginatus* females by the presence of *D. reticulatus*. In the inter-specific group, *D. marginatus* females attached for a longer time (mean 2.74 ± 1.12 h) than in the mono-specific group (mean 1.24 ± 0.97 h) (*p* < 0.0001). The feeding period of these females was shorter (9.45 ± 1.30 days) than in the mono-specific group (13.15 ± 2.53 days) (*p* < 0.0001), but they exhibited a statistically significantly higher body weight in comparison with the females from the mono-specific infestation (*p* = 0.0155). In *D. reticulatus* females*,* no significant difference was found in the host attachment and feeding rates between the mono-specific and inter-specific groups.

**Conclusions:**

The differences in the behaviour of the females from both species during co-feeding reflect physiological adaptation to environmental conditions, which enables them to ingest blood and reproduce. During co-feeding of *D. reticulatus* and *D. marginatus* on the same host, two inter-specific systems with different physiological features are formed, which may influence the transmission of tick-borne pathogens.

## Background

Among the representatives of the genus *Dermacentor* recognized worldwide, two species, i.e. *Dermacentor reticulatus* (Fabricius, 1794) and *Dermacentor marginatus* (Sulzer, 1776)*,* have a great epidemiological significance in Europe. The geographical ranges of these tick species are different, although in some regions of the southern and central part of the continent they may inhabit the same hygrophilic or xerophilic vegetation habitats depending on geographical location ([1, 2], Stanko, personal communications). Anthropopressure-induced environmental changes as well as climate changes may contribute to expansion of their occurrence range. Additionally, representatives of both tick species may be carried by migrating animals to areas inhabited by another species, where under favourable conditions, ticks can develop and attack a variety of animals.

*D. reticulatus* and *D. marginatus* ticks transmit numerous pathogens, e.g. Tick-Borne Encephalitis Virus, Omsk Hemorrhagic Fever Virus, Crimmean Congo Hemorrhagic Fever Virus, *Rickettsia slovaca*, *Rickettsia raoultii*, *Rickettsia sibirica* as well as *Coxiella burnetii*, *Francisella* spp., Bartonella spp., *Anaplasma phagocytophilum, Anaplasma marginale* and *Babesia* spp. thereby contributing to maintenance of foci of human and animal tick-borne diseases [1, 3–5]. Since both species parasitize the same domestic and wild-living animals ([1], Stanko, personal communications) and may infest humans [3, 6], pathogen transmission may proceed in various inter- and intra-specific systems. Hence, knowledge of the course of *D. reticulatus* and *D. marginatus* feeding on the same host has great epidemiological importance. Therefore, we investigated if, and in what ways, co-feeding may influence the parasitic phase of these species.

## Findings

### Methods

#### Tick hosts

The experiments were conducted on five tick-naive albino New Zealand rabbits (*Oryctolagus cuniculus*) with an average weight of 3–3.5 kg kept under standard laboratory conditions in accordance with the requirements specified by the ethics committee. Three animals were used for the study on *D. reticulatus* and *D. marginatus* ticks during a simultaneous infestation by inter-specific groups, and the other two rabbits were hosts in the examinations of a simultaneous mono-specific infestation of each of the tick species studied (control groups). The whole study was carried out in 2010.

#### Tick collection and rearing

Unengorged adult specimens of *D. reticulatus* were collected near Lublin in Poland and *D. marginatus* were found near Zádel in Slovakia during the spring activity peak. The common flagging method used consisted in sweeping the vegetation with a 1 m^2^ flannel cloth. In the laboratory, the sampled ticks were identified to species and gender using the keys of Siuda [1], and next transferred from glass transport containers to rearing chambers, in which 80 % humidity and temperature of 25 °C were maintained for *D reticulatus*, and 80 % humidity and 28 °C for *D. marginatus*. Such conditions had proved favourable for maintaining adult forms of the aforementioned ticks in previous investigations ([5], unpublished observations). Constant humidity values of 80 % were maintained using a saturated KNO_3_ solution in accordance with the method developed by Winston and Bates [7]. The experiments involved 100 females and 50 males of *D. reticulatus,* and 100 females and 50 males of *D. marginatus*.

In order to investigate the course of the parasitic phase, tick specimens were placed on rabbits’ back in a cloth bag attached to shaved skin, which prevented the parasites from spreading and ensured control over the experiment. Investigations of the rate of tick attachment and feeding were carried out at 20 ± 3 °C room temperature and 38 ± 1 % humidity.

#### Experimental groups

The dependencies between the tick species in the parasitic phase of the life cycle were investigated simultaneously in the following experimental groups:

- **infestations of two tick species (inter-specific infestations)** - 20 females and 10 males of *D. marginatus* and 20 females and 10 males of *D. reticulatus* on each host,
- **infestations of one tick species (mono-specific infestations)**- 40 females and 20 males of *D. marginatus* or *D. reticulatus* on each host.

#### Investigations of the rate of tick attachment and feeding

Prior to feeding, unfed *D. marginatus* and *D. reticulatus* ticks were weighed using an analytical balance with an accuracy of 0.0001 g. To distinguish between the species and gender of specimens, ticks were marked with different colours of oil markers. The behaviour of the ticks during the questing and attachment period was observed every 0.5 h until the time of strong attachment of their hypostomes to the host skin; after the beginning of feeding, the observations were held throughout the parasitic phase period at 24-h intervals at the same time of day. Immediately after the ticks detached from the host, engorged females were carefully collected from the host skin, weighed, and placed in rearing chambers.

Based on the results, the following parameters and indices of the parasitic phase were determined: attachment dynamics- percentage of specimens attached to host skin in a specified time, attachment period (AP), feeding period (FP), and female engorgement weight (FEW) defined in a previous paper by Buczek et al. [8].

#### Statistics

The Mann–Whitney *U* test was used in order to check whether there were significant differences in the parameter values between the species mono-specific and inter-specific groups. The calculations were completed in Statistica 5 PL and Microsoft Excel XP programmes.

#### Ethical approval

The study was performed with the full approval of Commission for Animal Experiments (ethical approval no 41/2006).

## Results

In the experimental conditions of temperature and humidity, *D. reticulatus* females attached to the skin within 0.5- 5 h after being placed on the rabbits (Table 1). No significant (*p* = 0.9898) difference was found in the length of the skin attachment period between *D. reticulatus* females in the mono-specific and inter-specific species groups.

**Table 1:** Parameters of the parasitic phase in *Dermacentor reticulatus* and *Dermacentor marginatus* females feeding on rabbits in mono-specific and inter-specific groups at 20 ± 3 °C and 38 ± 1 % RH

Species	Variable	Group	M	SD	Min.	Max.	Mann–Whitney *U* test
*Dermacentor reticulatus*	AP	mono-specific	2.60	1.43	0.50	5.00	0.9808
		inter-specific	2.66	0.98	0.50	4.50	
	FP	mono-specific	10.05	0.88	8.00	11.00	0.1202
		inter-specific	9.63	1.55	8.00	12.00	
	FEW	mono-specific	0.41	0.06	0.31	0.53	0.8361
		inter-specific	0.42	0.06	0.32	0.55	
*Dermacentor marginatus*	AP	mono-specific	1.21	0.97	0.50	5.00	<0.0001
		inter-specific	2.74	1.12	0.50	4.50	
	FP	mono-specific	13.15	2.53	10.00	17.00	<0.0001
		inter-specific	9.45	1.30	8.00	12.00	
	FEW	mono-specific	0.57	0.13	0.38	0.87	0.0155
		inter-specific	0.65	0.14	0.40	0.89	

*M* mean, *SD* standard deviation, *AP* attachment period (h), *FP* feeding period (days), *FEW* female engorged weight (g)

**Fig. 1:**
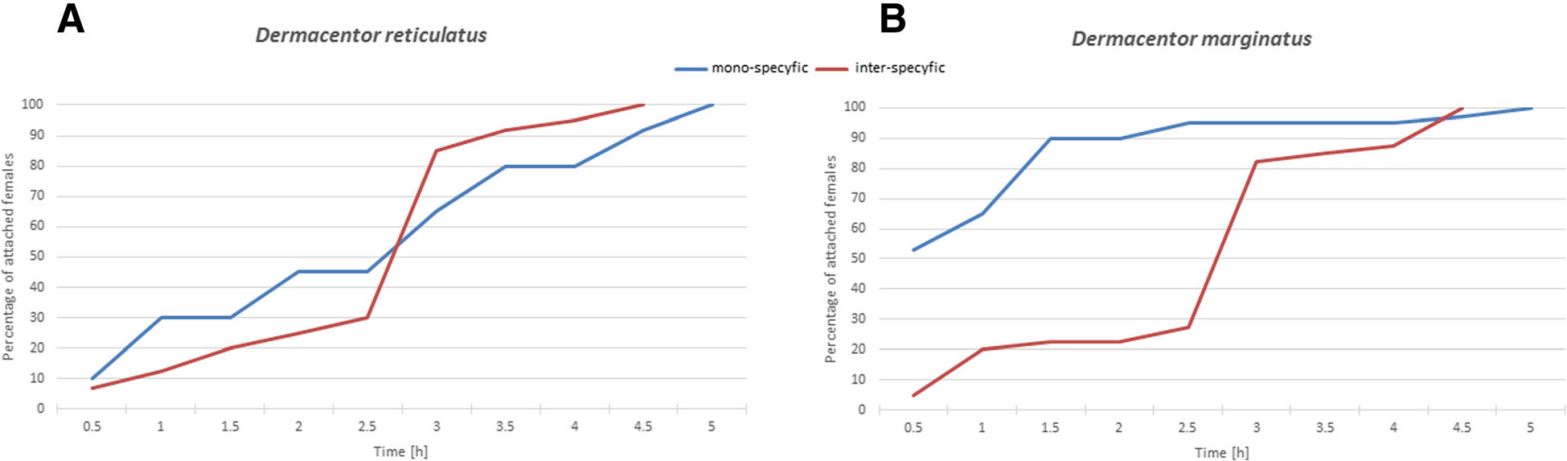
Dynamics of tick attachment to rabbit skin in the mono-specific and inter-specific species group; **a** *Dermacentor reticulatus* and **b** *Dermacentor marginatus*, at 20 ± 3 °C and 38 ± 1 % RH

The number of females that began feeding increased with the duration of the experiments; the largest numbers attached to rabbit skin within 1 – 3.5 h after being transferred on the host (Fig. 1a, b). The feeding dynamics differed between the mono-specific and inter-specific groups of *D. reticulatus* females (Fig. 1a). *D. reticulatus* females ingested blood longer exclusively in the presence of specimens from this species. When *D. marginatus* females were co-feeding with them, the mean length of the feeding period was shorter by 8.28 %. However, the length of the feeding period in both female groups, 10.5 ± 0.88 and 9.63 ± 1.55 days on average (Table 1), was not statistically significant (*p* = 0.1202). Similarly, the female engorgement weight did not differ significantly (*p* = 0.8361) between the homogenous and inter-specific groups of *D. reticulatus* ticks.

In turn, the feeding parameters in *D. marginatus* varied when adult stages of *D. reticulatus* parasitized the same host. In the inter-specific group, *D. marginatus* females attached within a longer time (mean 2.74 ± 1.12 h) than in the mono-specific group (mean 1.24 ± 0.97 h) (Fig. 1b). These differences were statistically significant (*p* < 0.0001). Similarly, the length of the feeding period exhibited statistically significant differences (*p* < 0.0001) between the groups. The feeding period in the case of *D. marginatus* females was longer in the homogenous (13.15 ± 2.53 days) than the inter-specific group (9.45 ± 1.30 days). *D. marginatus* females co-feeding in the inter-specific group with *D. reticulatus* specimens displayed higher body weight than in the mono-specific group (Table 1). The difference was statistically significant (*p* = 0.0155) in both groups.

## Discussion

Multiple environmental factors, primarily temperature [9] and photoperiod [10] as well as the physiological features of the host exert an effect on the course of tick attachment and feeding [11, 12]. Our studies have demonstrated that the presence of specimens of one species on the same host may affect the behaviour of representatives of another species. In *D. marginatus* females, competition with the other species led to a decreased rate of attachment to host skin and a shortened period of blood ingestion. However, feeding exhibited by the females from this species was more intensive than that displayed by the co-feeding *D. reticulatus* females. This type of behaviour reflects physiological adaptation of *D. marginatus* to conditions of limited access to the food source. Co-parasitism of various tick species on the same animals [13] and humans [14] has been reported in natural conditions. In Spain, 9 % of 3685 patients infested by ticks were attacked by two or a greater number of species [14].

Mating of Metastriata representatives takes place on the host; therefore, the prolonged period of attachment and the shortened feeding period in females of one species may constitute a biological barrier, efficiently preventing inter-specific crosses between ticks feeding on the same host. In the case of *D. marginatus* and *D. reticulatus*, the negative attempts at crossing the two species as well as results of molecular analysis have revealed their reproductive isolation [15]. Nevertheless, *D. marginatus* specimens with phenotypic traits of *D. reticulatus* have been found [16]. Furthermore, crosses between tick species from the genera *Dermacentor* yielded hybrids that were either unable to reproduce or were characterized by reduced survival [17].

Tick attachment is modified by aggregation-attachment pheromones (APP) released by other specimens feeding on the same host [18]. No investigations have been carried out on pheromones in *D. reticulatus* and *D. marginatus*, but differences in the response to the substances contained therein have been reported in other tick species [18]. In our experiments, we observed attachment of females and males from both *D. reticulatus* and *D. marginatus* species at a close distance to each other.

The differences in the feeding course in the tick species observed both in the mono-specific and inter-specific groups might be associated with the polymorphism of the proteins secreted by tick salivary glands [19]. Quantitative and qualitative differences in the composition of saliva of the particular tick species may stimulate different immune mechanisms in the host during the different phases of tick feeding.

Tick saliva contains many protein and lipid substances with a broad spectrum of pharmacological activity, which play an important role not only in the process of tick feeding, but also in the process of pathogen transmission by affecting the host immune system [20, 21].

The course of the parasitic phase in the ticks observed in our experiments was affected by the fact that the adult stages were feeding in aggregations, thus causing macroscopically visible lesions in the host skin. Severe cytological lesions and accumulation of inflammatory cells appeared in the histopathological image of skin sampled from the *D. reticulatus* feeding site [22], which, consequently, altered the composition of tick meal. Studies on the feeding course of *Rhipicephalus appendiculatus* species showed that the attachment and feeding rates were increased and the period of searching for a mate decreased during infestation with many specimens, compared with experiments with single tick pairs on the host [23]. Modification of feeding in one species induced by the presence of another species may lead to an increased rate of pathogen transmission, including saliva-activated transmission (SAT) [24], both in the inter-specific (tick-host) and intra-specific (tick-tick) systems. According to Richter et al. [25], the risk of infection with *Borrelia* spirochetes is six-fold greater when ticks co-feed with other infected specimens.

## References

[1] Siuda K. Ticks of Poland. Part II, Systematics and Distribution. Warsaw: PTP; 1993 (in Polish).

[2] Bursali A, Keskin A, Tekin S (2013). Ticks (Acari: Ixodida) infesting humans in the provinces of Kelkit Valley, a Crimean-Congo Hemorrhagic Fever endemic region in Turkey. Exp Appl Acarol.

[3] Földvári G, Rigó K, Lakos A (2013). Transmission of *Rickettsia slovaca* and *Rickettsia raoultii* by male *Dermacentor marginatus* and *Dermacentor reticulatus* ticks to humans. Diagn Microbiol Infect Dis.

[4] Bonnet S, de la Fuente J, Nicollet P, Liu X, Madani N, Blanchard B (2013). Prevalence of tick-borne pathogens in adult *Dermacentor* spp. ticks from nine collection sites in France. Vector Borne Zoonotic Dis.

[5] Šimo L, Kocáková P, Sláviková M, Kubeš M, Hajnická V, Vančová I (2004). *Dermacentor reticulatus* (Acari, Ixodidae) female feeding in laboratory. Biologia, Bratislava.

[6] Bursali A, Tekin S, Orhan M, Keskin A, Ozkan M (2010). Ixodid ticks (Acari: Ixodidae) infesting humans in Tokat Province of Turkey: species diversity and seasonal activity. J Vector Ecol.

[7] Winston PH, Bates DH (1960). Saturated solutions for control of humidity in biological research. Ecology.

[8] Buczek A, Lachowska-Kotowska P, Bartosik K (2015). The effect of synthetic pyrethroids on the attachment and host-feeding behaviour in *Dermacentor reticulatus* females (Ixodida: Amblyommidae). Parasit Vectors.

[9] Lees AD (1969). The behaviour and physiology of ticks. Acarologia.

[10] Belozerov VN, Obenchain F, Galun R (1982). Diapause and biological rhythms in ticks. Physiology of ticks.

[11] Carroll JF (2002). How specific are host-produced kairomones to host-seeking ixodid ticks?. Exp Appl Acarol.

[12] Bartosik K, Buczek A (2012). The impact of intensity of invasion of *Ixodes ricinus* and *Dermacentor reticulatus* on the course of the parasitic phase. Ann Agric Environ Med.

[13] Paulauskas A, Radzijevskaja J, Rosef O, Turcinaviciene J, Ambrasiene D (2009). Infestation of mice and voles with *Ixodes ricinus* ticks in Lithuania and Norway. Est J Ecol.

[14] Fernández-Soto P, Pérez-Sánchez R, Encinas-Grandes A, Alamo SR (2006). *Rickettsia slovaca* in *Dermacentor* ticks found on humans in Spain. Eur J Clin Microbiol Infect Dis.

[15] Zahler M, Gothe R (1997). Evidence for the reproductive isolation of *Dermacentor marginatus* and *Dermacentor reticulatus* (Acari: Ixodidae) ticks based on cross-breeding, morphology and molecular studies. Exp Appl Acarol.

[16] Estrada-Peña A, Estrada-Peña R (1992). Notes on *Dermacentor* (Acari: Ixodidae) ticks (IV): morphological covariation of *D. marginatus* (Sulzer). Acarologia.

[17] Oliver JH, Wilkinson PR, Kohls GM (1972). Observations on hybridization of three species of North American *Dermacentor* ticks. J Parasitol.

[18] Sonenshine DE (1991). Biology of ticks.

[19] Kazimírová M, Štibrániová I (2013). Tick salivary compounds: their role in modulation of host defences and pathogen transmission. Front Cell Infect Microbiol.

[20] Hajnicka V, Kocakova P, Slovak M, Labuda M, Fuchsberger N, Nuttall P (2000). Inhibition of the antiviral action of interferon by tick salivary gland extract. Parasite Immunol.

[21] Poole NM, Mamidanna G, Smith RA, Coons LB, Cole JA (2013). Prostaglandin E2 in tick saliva regulates macrophage cell migration and cytokine profile. Parasit Vectors.

[22] Buczek A, Kuśmierz A, Olszewski K, Buczek L, Czerny K, Lańcut M, Bernini F, Nannelli R, Nuzzaci G, de Lillo E (2002). Comparison of rabbit skin changes after feeding of *Ixodes ricinus* (L.) and *Dermacentor reticulatus* (Fabr.). Acarid phylogeny and evolution, adaptation in mites and ticks.

[23] Wang H, Hails RS, Cui WW, Nuttall PA (2001). Feeding aggregation of the tick *Rhipicephalus appendiculatus* (Ixodidae): benefits and cost in the contest with host responses. Parasitology.

[24] Jones LD, Matthewson M, Nuttall PA (1992). Saliva-activated transmission (SAT) of Thogoto virus: dynamics of SAT factor activity in the salivary glands of *Rhipicephalus appendiculatus*, *Amblyomma variegatum*, and *Boophilus microplus* ticks. Exp Appl Acarol.

[25] Richter D, Allgöwer R, Matuschka FR (2002). Co-feeding transmission and its contribution to the perpetuation of the Lyme disease spirochete *Borrelia afzelii*. Emerg Infect Dis.

